# High levels of consanguinity in a child from Paquimé, Chihuahua, Mexico

**DOI:** 10.15184/aqy.2024.94

**Published:** 2024-08-13

**Authors:** Jakob Sedig, Meradeth Snow, Michael Searcy, José Luis Punzo Diaz, Steven LeBlanc, Frank Ramos, Laurie Eccles, David Reich

**Affiliations:** 1) Department of Human Evolutionary Biology, Harvard University, Cambridge, MA, USA; 2) Department of Anthropology, University of Montana, Missoula, MT, USA; 3) Department of Anthropology, Brigham Young University, Provo, UT, USA; 4) Centro Instituto Nacional de Antropología e Historía-Michoacán, Instituto Nacional de Antropología e Historia, Mexico; 5) Peabody Museum of Anthropology and Ethnology, Harvard University, Cambridge, MA, USA; 6) Department of Geological Sciences, New Mexico State University, Las Cruces, NM, USA; 7) Human Paleoecology and Isotope Geochemistry Lab, Department of Anthropology, The Pennsylvania State University, PA, USA; 8) Department of Genetics, Harvard Medical School, Boston, MA, USA; 9) Howard Hughes Medical Institute, Harvard Medical School, Boston, MA 02446, USA; 10) Broad Institute of Harvard and MIT, Cambridge, MA, 02142, USA

**Keywords:** Ancient DNA, Paquimé, Sr Isotopes, Consanguinity, Social organization

## Abstract

This study reports results from the ancient DNA analysis of a unique child burial at Paquimé, northern Chihuahua, Mexico. Located between Mesoamerican and Ancestral Puebloan groups, Paquimé (also known as Casas Grandes) was a vibrant multicultural centre during the 13th-14th centuries AD. Archaeologists have long debated Paquimé’s social organization. Our analysis of Burial 23-8 has revealed that this child, placed under the centre post of an important room, had parents who were close genetic relatives. We argue that this child’s consanguinity and special depositional context resulted from an elite family’s practice of aggrandizing social status.

## Introduction

Though archaeologists have studied Paquimé (a UNESCO World Heritage Site in Chihuahua, Mexico) for decades, key debates remain unresolved, particularly concerning its social organization. Was it cosmopolitan? An elite-managed hierarchical society? Was its efflorescence the result of natural population growth or a large migration during the 12^th^-13^th^ centuries AD? In this study, we examine how Burial 23-8 (Reich lab ID# I22220) from the House of the Well, a genetically male child who had parents that were more closely related than first cousins, can start to provide answers to these questions.

## Paquimé background

**Figure 1.**
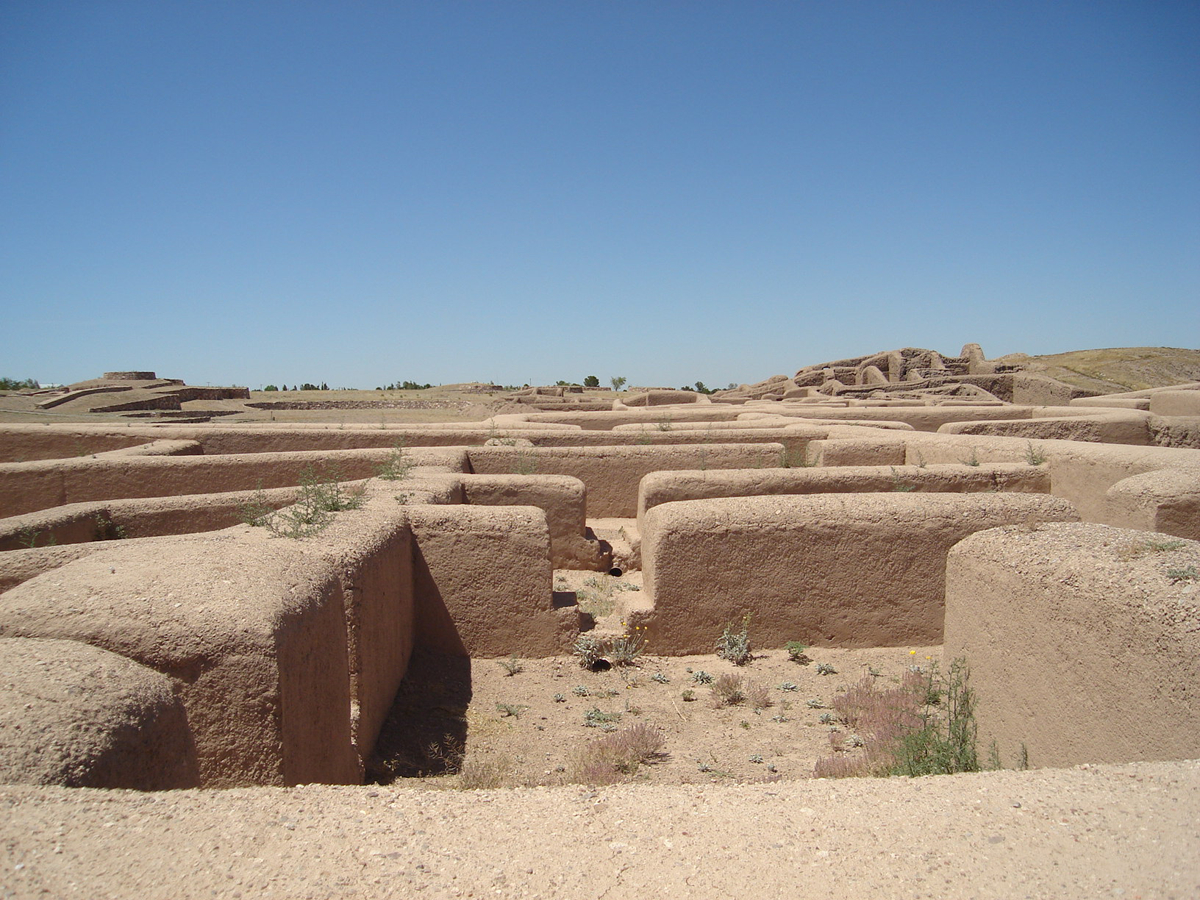
Adobe architecture at Paquimé.

Paquimé is one of the most significant sites in the Mexican Northwest/US Southwest (Northwest/Southwest). The Joint Casas Grandes project, led by Charles Di Peso, excavated a portion of the site from 1959–1961. The project and subsequent analyses form the foundation of archaeological understanding about Paquimé. Paquimé dates to AD 1200–1450 and was made primarily of adobe architecture (Figure 1). Its size and elaboration were unrivalled in the precontact Northwest/Southwest, and it was undoubtedly the political and ceremonial centre of the region. Paquimé had ~1100 rooms, and some of the apartment-style buildings were at least three stories tall. Multiple architectural features distinguish it from contemporaneous sites, including an approximately 113-meter-long “serpent” mound, a large cruciform mound, at least two ballcourts, plazas, an area with large agave roasting pits, and an aqueduct that carried water into the site from a spring approximately six kilometres to the northwest. Abundant trade goods, especially from West Mexico, were stored at Paquimé (including over four million pieces of marine shell) (Nelson *et al*. 2015; Punzo Díaz & Villapando 2016). Paquimé also was a macaw breeding community, with macaw pens and hundreds of macaw burials (Di Peso 1974).

Paquimé undoubtedly was central to socio-political developments in its region, though scholars continue to debate the nature of Paquimé’s origin, social organization, and decline. Archaeologists have revised many of Di Peso’s hypotheses, especially that Mesoamerican *pochteca* traders founded Paquimé (Dean & Ravesloot 1993; Whalen & Minnis 2009; Pailes & Searcy 2022). Scholars continue to debate whether Paquimé’s efflorescence was the result of migration from the Mimbres-Mogollon region to the north, influence by an elite lineage from the Chaco Canyon-Aztec Ruins region, local population growth, or allure of socio-religious leaders (Whalen & Minnis 2001a; b; VanPool & VanPool 2007; Lekson 2009; Pailes & Searcy 2022). There also is no consensus on Paquimé’s end, though evidence suggests it was unpleasant. Close to 100 human skeletons from the site’s terminal occupation were left unburied, which have been attributed to violence, disease, or starvation, raising the possibility that Paquimé as a regional urban centre met an ignominious end (Di Peso 1974a; Ravesloot 1988; Walker 2002; Casserino 2009). Despite this, some Paquimé residents and their descendants remained in the region after Paquimé’s demise and moved into the surrounding area. Through a reanalysis of Spanish documents, extant Colonial-era architecture, artifacts, and cranial modification, Douglas and Brown (2023) argue that the Suma people, whom the Spanish encountered during their early expeditions, were descendants of the inhabitants of Paquimé and the Casas Grandes regional system.

### Insights into Paquimé’s Hierarchy from Burial Data

Paquimé’s social structure, degree of hierarchy, and elite influence have received much attention since the Joint Casas Grandes project (e.g., Schaafsma & Riley 1999; Whalen & Minnis 2001a; c; VanPool & VanPool 2007; Lekson 2009; Pailes & Searcy 2022). Studies that have explored questions of the site’s social structure through burial data (e.g., Ravesloot 1988; Casserino 2009; Rakita 2009; Offenbecker 2018; Waller et al. 2018) have found evidence of social stratification and concluded that some lineages were linked to political and religious power (Pailes and Searcy 2022:90). Rakita (2009) argues for the presence of an exclusive ancestor worship cult at Paquimé that legitimized power through sacrifice (2009:148). Other studies of Burial group 44–13 in the House of the Dead (Offenbecker 2018; Waller et al. 2018), the most elaborate burial at Paquimé, found social distinctions among 12 individuals split between upper and lower layers. The upper layer contained the co-mingled skeletal remains of five individuals who had indicators of poor health, such as enamel hypoplasia and hypoplastic lesions, along with cut marks and evidence of post-mortem processing (including possible cannibalism; Casserino 2009). In contrast, the seven individuals in the lower layer were articulated and associated with ritually significant grave goods (e.g., smashed ceramic hand drums, a turkey sacrifice, and Ramos Black ceramic vessels). They also had many fewer markers of health stress and post-mortem processing. Offenbecker (2018) conducted a Sr isotope study of three individuals from the upper layer and two from the lower and found that the lower layer individuals were locals, while two of the three individuals from the upper layer were non-local. Additionally, King *et al*. (2017) identified microscopic botanical evidence embedded in the dental calculus of two of the individuals in this burial that suggested they had recently consumed corn beer and may have been inebriated shortly before their deaths and interment. Taken together, these lines of evidence could be indicative of sacrificial victims being brought from outside Casas Grandes, ritually killed, and then buried with elites who were born and lived locally in the region.

### House of the Well, Unit 8, Burial 23-8

**Figure 2.**
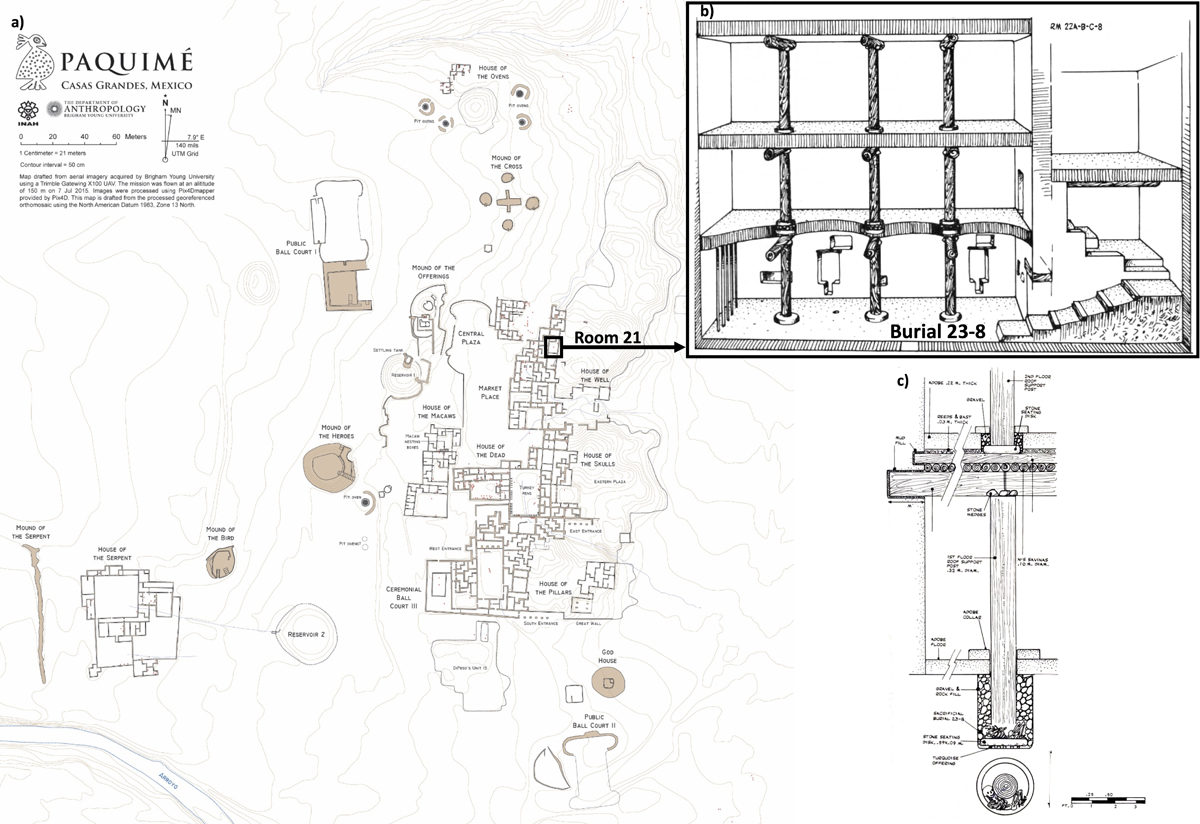
Location of Room 21 and Burial 23-8 at Paquimé. A) Paquimé site map, produced by M. Searcy. B) Diagram of room 21.Adapted from Di Peso et al. 1974 volume 4, pg 408. Courtesy of The Amerind Foundation, Inc., Dragoon, Arizona. C) Position of Burial 23-8. From Di Peso et al. 1974 volume 4, pg. 410. Courtesy of The Amerind Foundation, Inc., Dragoon, Arizona.

Located in the northeast portion of Di Peso’s excavation (Figure 2a), the House of the Well (Di Peso’s Unit 8) was given its name due to the unique subterranean walk-in well found within it (Di Peso 1974 vol 2:382). This well was approximately 12 meters underground (Di Peso 1974 vol 2:377). To access it, one had to use a steep staircase, passing a human calvarium (skull) embedded in the floor entrance and other ritual artifacts such as copper tinklers, small stone effigies, and turquoise and shell beads (VanPool 2003:702). According to Di Peso (1974 vol 2:382), the habitations closest to the well were affluent and had “the best acequia and sewage services.” The rooms in the House of the Well also contained more specialized goods than any other area of the site, with millions of marine shells from 60 species, the bulk of the raw ricolite, turquoise, salt, selenite, and copper ore, as well as a stack of 50 or more non-locally made Gila Polychrome bowls, smoking pipes, decapitated effigy vessels, and the highest number of macaw sacrifices (n=34) of any area at Paquimé (Di Peso *et al*. 1974; Offenbecker 2018; VanPool 2003). Through an analysis of ritual objects, particularly ceramic effigy vessels and imagery, smoking pipes, and caches of mineral concretions, quartz, and small fetishes, VanPool (2003) argues that shaman-priests were elite leaders at Paquimé and that their power was concentrated at the House of the Well.

Though not in direct contact with the well itself, Room 21c in the House of the Well was the bottom floor of a rare three-story room that was an important, non-typical domestic room. It lacked a cooking hearth, was larger than most rooms (12.75m × 5.35m), had elaborate stairs to the upper story, artefacts including copper objects, and likely a scarlet macaw pen on an upper floor. Three substantial wooden pillars supported the roof and upper floor (Figure 2b). Each pillar posthole had large, shaped stone support discs at the bottom, with turquoise pendants placed as offerings beneath them. Wrapped around the base of one roof-support post was Burial 23-8, which consisted of a two- to five-year-old child placed on top of a sandstone disc, underneath of which were found five turquoise bead pendants (Figure 2c). According to Di Peso *et al*. (1974:376), “the body position of Burial 23-8 indicated that this child was placed as some sort of supplicatory offering at the time the post support was seated, during the Paquimé Phase remodelling.” Additionally, the back left side of the skull exhibits damage, possibly from a fatal blow. Though Di Peso recorded several other possible sacrifices, most notably the upper-level individuals in Burial 44–13 located in the House of the Dead, Burial 23-8 was unique in its form and context. The Joint Casas Grandes Project excavated 57 individuals from the House of the Well (9.9% of the burials from the site). Of those 57 individuals, 25 were in formal pits beneath house floors, 18 were in formal pits beneath plazas, and 14 were unburied. DiPeso excavated thirteen children in the House of the Well, with six beneath house floors, four beneath plazas, and three unburied. Interred with turquoise pendants, Burial 23-8 was the richest burial in the House of the Well. According to DiPeso *et al*. (1974:376), only 12 House of the Well burials had funeral furniture, the most common item consisting of “a single ceramic vessel.” These combined factors led Di Peso and subsequent researchers to classify Burial 23-8 as a sacrifice. Our new genetic analyses, described below, reveal another distinct attribute of this child interment.

## Genetic Analysis of Burial 23-8

Burial 23-8 was analysed as part of the Proyecto de Investigación de Poblaciones Antiguas en el Norte y Occidente de México (PIPANOM), which examines changes in population structure through time in western and northern Mexico, in close collaboration with researchers in the region (see Ethics Statement). Our analysis pipeline for a sample from Burial 23-8 included processing of skeletal material, sequencing, bioinformatics, and data quality assessments (as described, for example, in Lazaridis *et al*. 2022; see Supplementary Material for methods description). These methods confirmed that Burial 23-8 produced authentic aDNA data (Table 1; Supplementary Material; Supplementary Table S.1). DNA sequences have appreciable damage at the terminal ends as expected for genuine aDNA. The match rate to the mitochondrial consensus sequence has a 95% confidence interval of 99.4–100%, consistent with minimally contaminated data (Supplementary Table S.2). Uniparental markers (mtDNA haplogroup C1b and Y-chromosome haplogroup Q1a2a2b1a~) are consistent with Native American populations.

After confirming data authenticity, we combined Burial 23-8’s genetic data with that of 609 previously published ancient individuals from across the Americas (Supplementary Dataset 1) and 170 previously published modern individuals (including Chane, Huichol, Karitiana, Zapotec, Mixe, Mixtec, Piapoco, O’odham, Quechua, Surui, European, and Mbuti; Supplementary Dataset 2). We conducted population-level analyses (Principal Components Analysis (PCA) (Patterson *et al*. 2006), ADMIXTURE (Alexander *et al*. 2009), and outgroup f_3_ statistics (Patterson *et al*. 2012)) to examine Burial 23-8’s ancestry and if it was significantly different from that of other previously published individuals.

**Figure 3.**
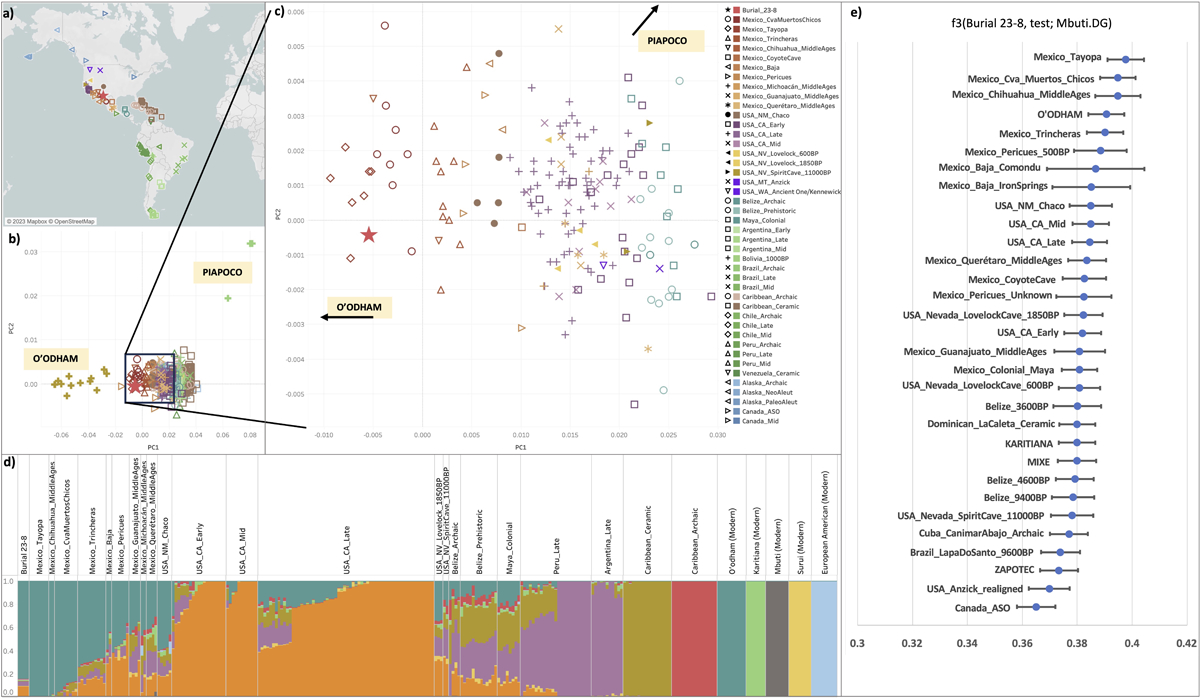
Population genetics analysis of Burial 23-8. A) Map showing location of all published Americas individuals included in analysis. Map generated in Tableau version 2023.1 using OpenStreetMap data. B) PCA of Burial 23-8 (red star) with published modern and ancient individuals from the Americas. PCA created by calculating axes using modern populations and projecting ancient individuals onto the axes (Supplementary Data 2). C) Zoom-in of PCA with only Meso- and North America individuals. D) ADMIXTURE plot at K=9 of BURIAL 23-8 (far left) with other published ancient (left) and modern (right populations). Populations ordered by geography and similarity of admixture proportions to Burial 23-8. E) Outgroup f3 results of test f3(Burial 23-8, Test Population; Mbuti.DG). Higher values indicate greater amounts of allele sharing with Burial 23-8. Error bars represent two standard errors. Modern populations capitalized.

PCA analysis (Figure 3b–c) demonstrates that Burial 23-8 and other previously published ancient individuals from the Northwest/Southwest (Villa-Islas *et al*. 2023; Nakatsuka *et al*. 2023) are shifted towards modern-day O’odham (Pima) ancestry (modern O’odham people are Uto-Aztecan speakers and maize farmers whose homeland traditionally stretched across the modern US-Mexico border). The O’odham were one of the groups the Spanish encountered during their conquest of the Northwest/Southwest beginning in the mid-16^th^ century and still reside in the region today.

Outgoup f3 analysis (Figure 3e) provides similar results to PCA, with Burial 23-8 having more allele sharing with modern O’odham and ancient individuals geographically closest to Paquimé. As with PCA and outgroup f_3_ analysis, ADMIXTURE analysis reveals that Burial 23-8 has ancestry most like ancient individuals in closest geographic proximity (Figure 3d). Similarity in ancestry decreases with increasing distance from Paquimé.

Our analyses indicate that Burial 23-8’s ancestry was like that of other individuals who lived in the Northwest/Southwest during the last 2000+ years, with Burial 23-8’s ancestry particularly close to modern O’odham. Though we do not explore substructure within this ancestry, we do note that in general the ancestry of ancient individuals in Central and North America is clinal and correlates with geography; in other words, individuals in closest geographic proximity are genetically more related to each other, with decreasing relatedness as geographic distances increase. This finding replicates previous studies of ancient and modern populations in Central and North America (Aguilar-Ordoñez *et al*. 2021; Villa-Islas *et al*. 2023).

### Runs of Homozygosity

Runs of homozygosity (RoH) are duplicate segments of an individual’s genome, resulting from inheriting homozygous segments from closely related parents (Ceballos *et al*. 2018). Quantifying and analysing RoH in ancient genomes can be used to estimate the size of the communities in which people lived and the degree of relatedness (consanguinity) of an individual’s parents (e.g., Skourtanioti *et al*. 2020; Cassidy *et al*. 2020; Ringbauer *et al*. 2020). RoH is recorded in centimorgans (cMs), which is a measure of genetic distance between two positions on a chromosome. Higher cM values demonstrate that more of the genome is held in common between two (or more) ancestral individuals. Analytical tools such as *hapROH* (Ringbauer *et al*. 2021) quantify the RoH found in an ancient genome. Segments of RoH are pooled by length and summed. The total sum of short-medium RoH segments is used to make inferences about population/mating pool size, as small, isolated populations are likely to contain individuals that are distantly (or closely) related to one another and, thus, mating pairs in the population will likely share some shorter lengths of RoH (i.e., more closely related by chance). Conversely, if these individuals had low summed short-medium RoH values, their immediate ancestors were likely part of larger, more heterogeneous populations. Summed RoH values >20cM (henceforth long RoH) can be used to make inferences about consanguinity. The more closely related an individual’s parents were, the more that person’s genome will contain long RoH segments that span >20cM.

**Figure 4.**
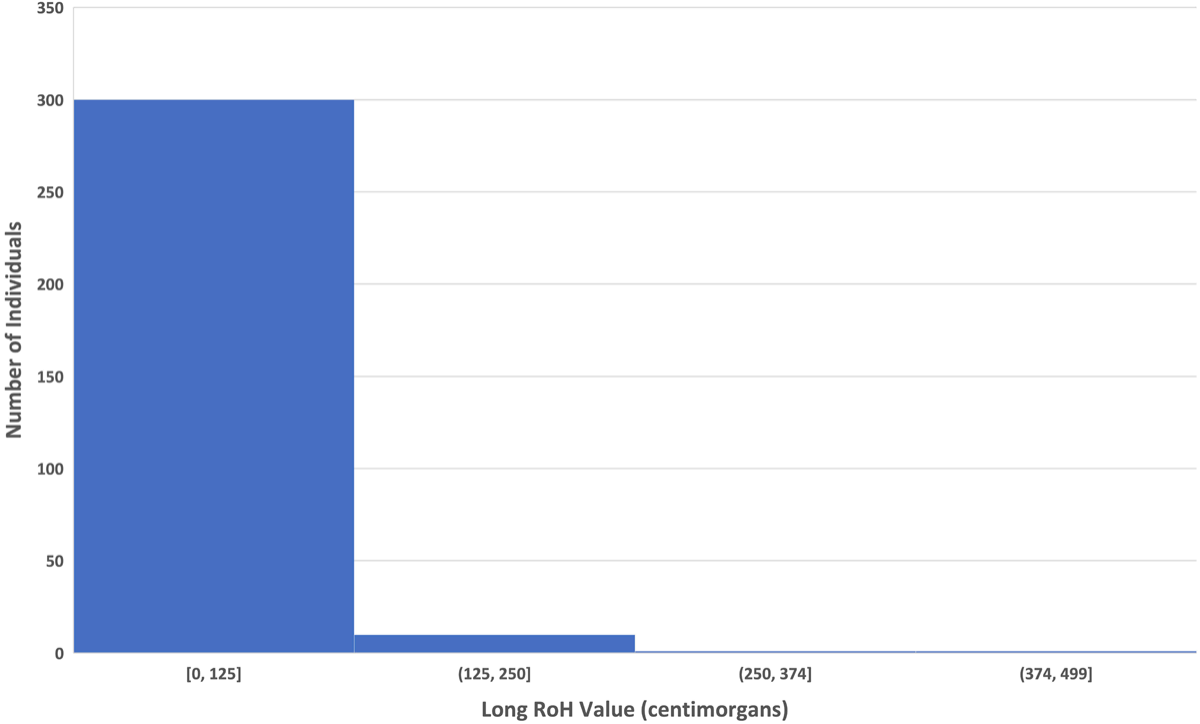
Histogram of long RoH for published Americas individuals binned by summed long RoH values.

**Figure 5.**
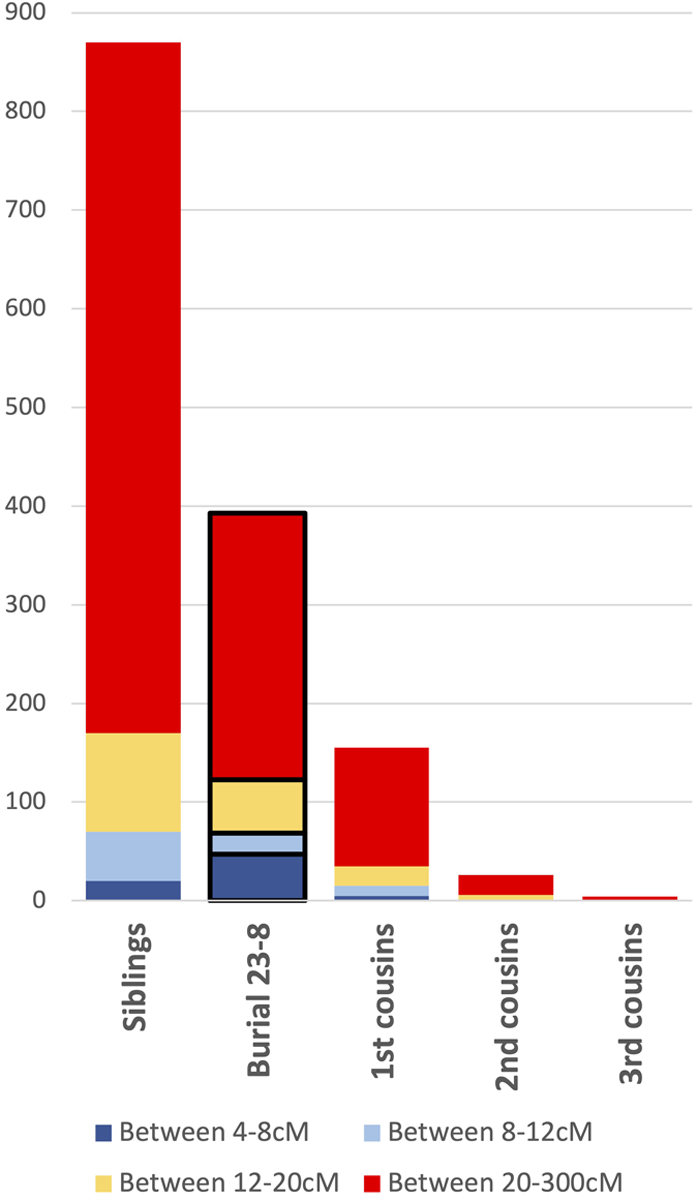
Stacked bar plot of RoH values for Burial 23-8 and expected values for specific familial relationships.

We found a long RoH value of 270.09 for Burial 23-8 using *hapROH* (Table 2). The mean and median long RoH values for 237 previously published Americas individuals for which coverage was high enough to calculate RoH are 17.76 and 0, respectively; Burial 23-8’s value of 270.09 is the largest of all previously published individuals from the Americas, except for a recently published individual from Santa Clemente Island, California (Table 3, Figure 4, Supplementary Dataset 3). In a simulation of 1000 individuals, Ringbauer *et al*. (2021) found that the range of long RoH for first cousins (3^rd^ degree genetic relatives) was between 50–500 cM (Ringbauer et al. 2021 Supplementary Material pg 32). However, only 29/1000 of these first cousin simulations had a value higher than 270.09, which suggests that it was unlikely that Burial 23-8’s parents were first cousins and that they were more closely related. We used data from Ringbauer *et al*. 2021 to compare the RoH values for Burial 23-8 with expected RoH values for particular familial relationships (Figure 5). The most probable type of relationship is second degree, which arises when parents are half-siblings, uncle-niece, aunt-nephew, or grandparent-grandchild (Ringbauer personal communication 2023).

### Radiocarbon analysis

**Figure 6.**
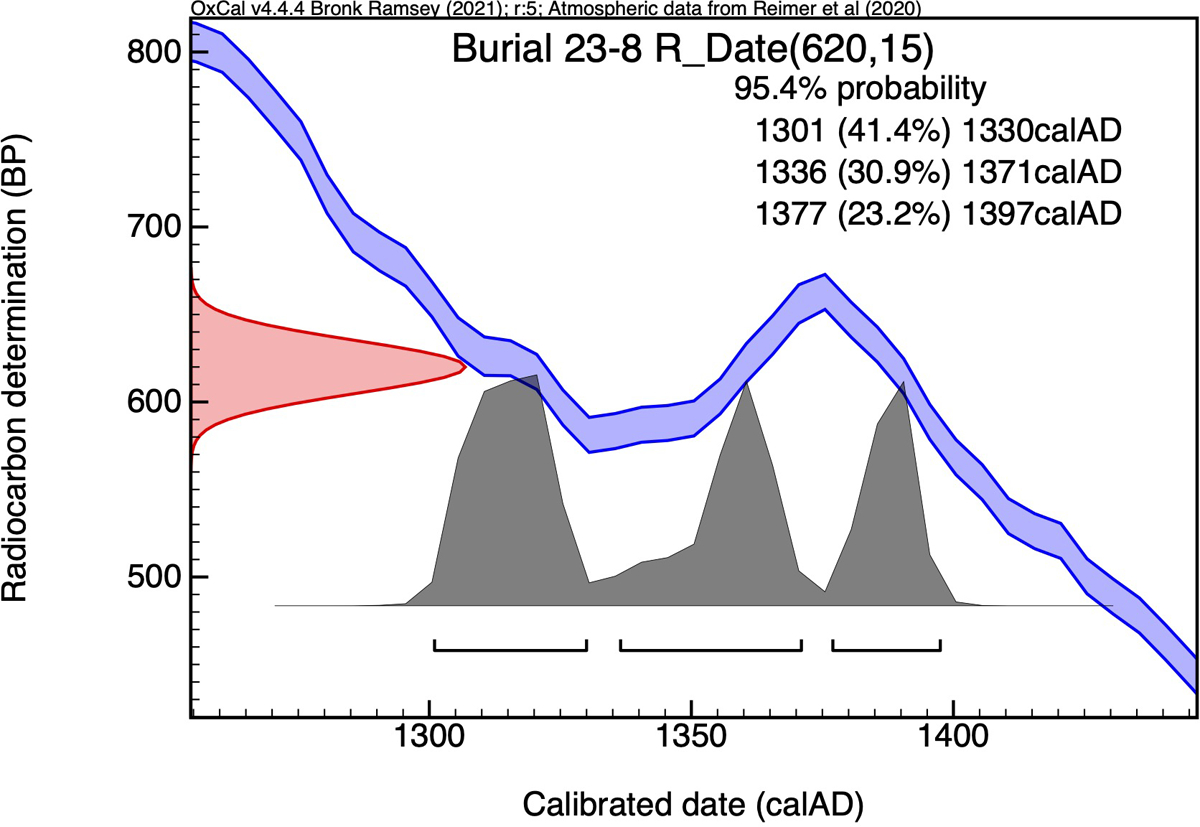
Radiocarbon age of Burial 23-8. Calibrated in OxCal v4.4.4 (Bronk Ramsey 2021), with IntCal 20 (Reimer et al. 2020).

We conducted additional biomolecular analyses to help contextualize the life of Burial 23-8. Radiocarbon dating produced a range of AD 1301–1397 (2σ; 620±15 BP; PSUAMS-10865; Figure 6, Supplementary Material), falling within Paquimé’s apogee during the Medio period. Di Peso *et al*. (1974b:407) produced a dendrochronological date of AD 1113p–1234vv for the post Burial 23-8 was wrapped around, predating the radiocarbon date we obtained by over a century. However, previous work has identified issues with Di Peso’s tree ring dates, mainly that many dates were too early because they did not account for the shaping of beams and removal of outer rings (Dean & Ravesloot 1993). It thus is likely that Burial 23-8’s interment was contemporaneous with the beam’s erection.

### Strontium analysis

**Figure 7.**
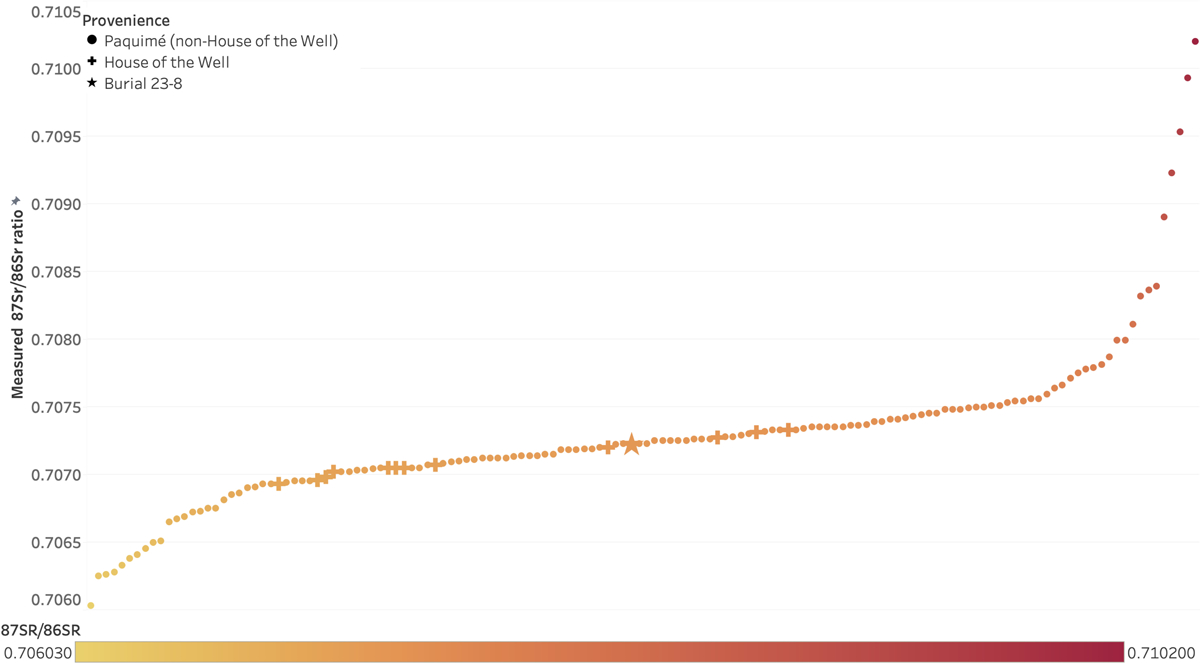
Strontium isotope values of Paquimé burials. All values other than Burial 23-8 (star) published in Offenbecker (2018:62–66).

We used remnant cochlea powder from ancient DNA analysis to analyse Burial 23-8’s Sr isotope ratio (Supplementary Material). Burial 23-8 yielded a ratio of 0.70723 ± 0.000010, falling within the range for the site (0.7068–0.7075; Figure 7) and suggesting the child spent his^1^ short life at or near Paquimé. Our Sr analysis aligns with Offenbecker’s (2018) previous isotopic research at Paquimé that found only local individuals buried in the House of the Well. This, coupled with the abundance of ritually significant features and artifacts found there, indicated to Offenbecker (2018) that the people buried in the House of the Well were part of a local elite group.

## DISCUSSION

Researchers have used RoH to study aspects of the past that were previously difficult to ascertain. Fernandes *et al*. (2020) used pools of 12–20 cM segments to examine ancient Caribbean population size. Interestingly, population estimates for two major clades, Ceramic-era southeast coast of the Dominican Republic and Ceramic-era Eastern Greater Antilles, were much smaller (Ne of 3082 (95% confidence interval 1530–8150)) than most previous estimates. Fernandes *et al*. (2020) also found that ancient inhabitants of the Caribbean did not have high levels of consanguinity overall; in other words, an individual’s parents were not likely to be more closely related than second cousins.

In contrast to the Caribbean, Ringbauer *et al*. (2020) found high levels of consanguinity after AD 1000 in the central Andes. The ancient individuals in the study had long RoH values typical of first- or second-degree cousins. Interestingly, the rate of close-kin unions was not consistent through time, increasing from 9% to 46% after AD 1000. The authors attribute this increase to changing kinship patterns during the Late Intermediate period after Tiwanaku and Wari cultures declined.

In a study examining RoH in a substantial global aDNA dataset (1785 individuals across 45,000 years), Ringbauer *et al*. (2021) found lower levels of consanguinity than might be expected from ethnographic/anthropological literature. Ringbauer *et al*. (2021) found that only 54/1785 ancient individuals had long ROH summing to above 50cM (with 11/54 individuals from isolated island populations). When Ringbauer *et al*. applied *hapROH* to modern data, which included approximately 150 present-day populations, they found that 176/1941 individuals had long RoH summing to >50cM (consistent with being 3^rd^–4^th^ degree relatives). These individuals geographically cluster in the present-day Near East, North Africa, Central/South Asia, and South America, where cousin marriages are more common than in other areas (such as Western Europe).

Cassidy *et al*. (2020) examined 44 Irish Meso- and Neolithic individuals using a different method to measure homozygosity in a genome. Their study found an instance of an individual from the Newgrange passage tomb with extreme consanguinity. This individual (NG10), an adult male, had parents who were most likely siblings (we computed a long RoH value of 687.41 for NG10 using *hapROH*). Cassidy *et al*. (2020) suggest that the pairing of NG10’s parents was socially sanctioned and likely restricted to ruling families to consolidate and legitimate power. Burial 23-8 has less long RoH than NG10; however, when considered with his unique burial context, the coupling of Burial 23-8’s closely related parents may also have been a way to legitimize elite hierarchical status.

### Consanguinity and social status

Ethnographic and sociological work has found that close-kin relationships are rare and that most social groups view unions of siblings as incest and thus taboo (Levi-Strauss 1969; Burton 1973; Schneider 1976; Shepher 1983; Bateson 2004; Gates 2004; Wolf & Durham 2004). There are exceptions to the nearly universal sibling incest taboo, perhaps the most well-known being sibling pairings in Roman Egypt (Scheidel 2005). A cross-cultural study by Struevant and Goggins (1964) found that taboos against sibling unions most often occurred in societies with some degree of social hierarchy and in which elites/rulers were exempt from social norms. Responding to Struveant and Goggins (1964), van den Berghe and Mesher (1981) found that 34/42 ethnographically observed societies condoned sibling or half-sibling mating only for royals or aristocrats. They state that “sibling incest is thus confirmed to be overwhelmingly a strategy of polygynous, high-status people; the higher the status, the more polygynous, the more likely the incestuous strategy” (van den Berghe and Mesher 1981:1988). Gates (2004) further expounds on the work of Struveant and Goggins (1964) and Berghe and Mesher (1981), arguing that sibling and other close-kin unions amongst elites in “high-chiefdom/almost-state” societies would have been a way for precocious leaders to “construct auras of power by haughty taboo-breaking” (2004:153).

Most of the anthropological and ethnographic discussion of close-kin union taboos and exceptions to them have focused on siblings. However, genetic studies suggest other types of close-kin relationships were also avoided. Of all the ancient individuals examined by Ringbauer *et al*. (2021), only two—an individual from Israel dating to the sixth millennium BC and an individual from Russia dating to the third millennium BC—had long RoH values greater than Burial 23-8 (545.02 and 324.87, respectively). Ringbauer *et al.’s* (2021) global aDNA study did find some modern individuals with long RoH over 50cM, though only 71 of 1941 had summed long RoH >120cM, the expected value for first cousins. This suggests that even first-cousin pairings (and anything closer than that) are typically taboo. Ringbauer and colleague’s (2020) Andean study provides a contrast to this, however, as multiple individuals had long RoH values expected for the offspring of first- or second-degree cousins (though only 2/13 individuals with elevated ROH had values expected for first-cousin unions). It is also worth noting that the small-scale polities of the Andean Late Intermediate Period had social hierarchy equivalent to the “high-chiefdom/almost-state” level societies discussed above that used close-kin relationships as political capital.

To summarize, our review of ethnographic/anthropological literature and global aDNA data suggests that taboos against close-kin unions were common and not limited to sibling relationships. The recorded instances of when relationships are condoned most often occur among elite families within socially stratified societies. These results suggest that Burial 23-8 was part of a similarly hierarchical society at Paquimé.

### Implications of Burial 23-8 for Paquimé

Burial 23-8’s parents were half-siblings, aunt/uncle-niece/nephew, grandparent-grandchild, or some other combination that shared between ~25–50% of their genomes. Only a few sequenced ancient individuals in the Americas (and globally) have long RoH values as high as Burial 23-8. It is, of course, possible that Burial 23-8 was the result of an illicit relationship or coerced sexual interaction. It is also possible that Burial 23-8’s parents were unaware of their close genetic relationship. Though it is impossible to know with certainty how Burial 23-8’s related parents interacted sexually to produce this genetically male individual, the unique burial context of Burial 23-8 in the House of the Well and his local Sr isotopic signature indicate that his parents did have special social standing, which aligns with studies that have found close-kin unions were primarily limited to high-status individuals who reproduced with relatives to consolidate power.

Rakita (2009) has argued that Paquimé’s elite used mortuary rituals and manipulation of skeletal remains with restricted access (such as those in Burial 44–13) to extend their status as people who could operate outside of social norms, thus codifying their elevated social positions. In the case of Burial 44–13, sacrificial victims were non-local, in poor health, processed (and possibly consumed), and not buried with any grave goods. Thus, their sacrifice starkly contrasts with Burial 23-8, who was buried near turquoise pendants, under a centre post in a ritually significant room, in an important building where only locals were buried, and who had parents that were closely related.

Unlike the sacrificial victims in the upper level of Burial 44–13 at the House of the Dead, Burial 23-8 was articulated and a local. His burial in the House of the Well, which previous studies have shown to be a ritually charged and significant place where local elites established and nurtured their power, suggests that he was part of the local lineage that may have controlled access to the well and other rituals at Paquimé (particularly related to water; Rakita 2009; VanPool 2003). As far as we know, there are no other recorded instances of burials with ritually significant grave goods placed under posts in the greater Northwest/Southwest. Child sacrifice was a practice that occurred throughout ancient Mesoamerica (Crandall & Thompson 2014; Toyne 2015; Prieto *et al*. 2019; Tiesler *et al*. 2021), as was the use of sacrifice in the consecration of ritually significant buildings (Medrano Enríquez 2021). In addition, studies of Precontact Mesoamerican rituals have shown that human sacrifice was one of the most potent ways to placate the gods or receive their assistance, with elite sacrifice being the most powerful (López Luján, Leonardo & Olivier 2010). Sacrificing a child born of two people from a local, elite lineage would have been a powerful way to consecrate the House of the Well and augment social, political, and ritual standing.

## Conclusion

This study is the first to report genome-wide data from Paquimé. These data provide exciting new insight into Paquimé’s social structure. Burial 23-8 at Paquimé was a young boy whose parents were closely related; indeed, his summed long RoH is the second highest of any ancient individual from the Americas published to date. He was the only individual at Paquimé found buried in a posthole. We find it very unlikely that these were coincidences. Archaeologists have long debated the nature of Paquimé’s social organization. The data from Burial 23-8 suggest that there was an elite class at Paquimé, who may have tried to consolidate power by establishing mating pairs of close relatives.

## Supplementary Material

Supplementary Material

Supplementary Dataset

## Figures and Tables

**Table 1—:** aDNA data generated for Burial 23-8

# 1240k SNPs	Autosomal Coverage	Genetic Sex	Mt haplogroup	Y-haplogroup
395547	.28x	M	C1b	Q1a2a2b1a~

**Table 2.** *hapROH* results for Burial 23-8 by RoH segment length

	# RoH segments	sum RoH
Between 4–8cM	9	47.01
Between 8–12cM	2	21.53
Between 12–20cM	3	54.08
Between 20–300cM	8	270.09

**Table 3.** Americas individuals with the highest measured long RoH

Individual ID # and Location	Sum total ROH segments >20cm
I0748 (California)^1^	499.06
**Burial 23-8 (Paquimé)**	**270.40**
I5320 (Alaska)^2^	248.39
SN-44 (California)^3^	247.85
I11968 (California)^1^	241.17
SN-11 (California)^3^	226.22
I0308 (Argentina)^4^	162.07
I13322 (Caribbean)^5^	137.28
I23708 (California)^1^	133.78
I0042 (Peru)^6^	133.77
I7966 (Caribbean)^5^	130.04
I11557.SG (California)^1^	108.32
I10758 (Caribbean)^5^	96.31

(1. Nakatsuka *et al*. 2023; 2. Flegontov *et al*. 2019; 3. Scheib *et al*. 2018; 4. Posth *et al*. 2018; 5. Fernandes *et al*. 2021; 6. Nakatsuka *et al*. 2020). Note: Expected summed long RoH values (from Ringbauer et al. 2021): Siblings: 625 cM, first cousins: 125 cM, second cousins: 30 cM, third cousins: 15 cM.

## Data Availability

Genomic data for Burial 23-8 are available at the European Nucleotide Archive and the National Centre for Biotechnology Information under the accession number PRJEB71964 and at https://reich.hms.harvard.edu/datasets.

## Footnotes

Ethics Statement Burial 23-8 was analysed as part of the joint PIPANOM project, of which authors M. Snow, JLPD, SL, DR, and JS are collaborators. Approval of the project was granted by the Instituto Nacional de Arqueología e Historia, no. 401.1S.3–2018/1732. Research and sampling were conducted using methods that minimize the destructive impact on skeletal material. Because of its Colonial history, consultation with local and Indigenous communities in Mexico is complex, and there is no standardized protocol beyond research approval by INAH. For this project, aDNA data and preliminary results generated in the United States were shared directly with Mexican collaborators and students and at academic and public symposiums on multiple occasions. We incorporated feedback from these sessions into ongoing PIPANOM research. This project contributed to capacity building in Mexico by using grant funding to purchase sampling equipment and laboratory supplies, providing training and information sessions to students and local community members, and supporting student stipends. This project also included the preparation of a Frequently Asked Questions (FAQ) document in Spanish that was distributed and made available at the Museo de las Culturas del Norte in the archeological site of Paquimé, Casas Grandes, Chihuahua, México.

1) We use “his” to refer to the male genetic sex of Burial 23-8, and acknowledge that we do not know the gender of this individual and how they identified while alive.
