# Supplementary material for: High levels of consanguinity in a child from Paquimé, Chihuahua, Mexico
Source: Antiquity. Author manuscript; Available in PMC 2024 Nov 1. (PMC11529752; doi:10.15184/aqy.2024.94)
Supplement: Supplementary Material [file NIHMS1959625-supplement-Supplementary_Material.docx]

**Ancient DNA laboratory work**

Ancient DNA from Burial 23-8 was collected as part of the Reich and Snow Labs’ collaboration and the Proyecto de Investigación de Poblaciones Antiguas en el Norte y Occidente de México (PIPANOM). Under the collaboration agreement, individuals from Paquimé to be sampled for ancient DNA were brought to the Snow Laboratory (University of Montana) where they underwent initial processing. This involved producing powder from petrous/cochlea or teeth; the powder was then divided with half retained by the Snow lab for furthering processing to obtain mtDNA data and the other half sent to the Reich lab for processing to obtain genome-wide ancient DNA data. The Reich lab received 0.05 grams of cochlea powder for processing; we describe the procedure below.

The powder that arrived at the Reich lab was turned into an extract through a series of previously described steps to isolate, clean, and amplify the DNA (Dabney *et al.* 2013; Rohland *et al.* 2015). In brief, the DNA was converted into a form that could be sequenced using a double-stranded library preparation protocol, which involved a partial pre-treating with the enzyme Uracil-DNA Glycosylase (UDG) to reduce the characteristic cytosine-to-thymine errors in ancient DNA at all but the terminal positions in the reads. Following Fu et al. (2013, 2015), approximately 1.2 million SNPs were targeted, sequenced on a HiSeq X10 Illumina instrument, and mapped to the human genome reference sequence hg19 as previously described (Mathieson *et al.* 2015). For the whole genome data, an allele at each position was determined by randomly sampling a single position. For the mitochondrial DNA, a consensus sequence was built aligning to the RSRS genome (Behar *et al.* 2012).

Sequencing statistics are presented in Supplementary Table 1 (below).

*Contamination estimation*: We used several methods to assess evidence of contamination for Burial 23-8. We measured the rate of damage in the first nucleotide and found that Burial 23-8 did not have less than 3% cytosine to thymine substitution rate in the first nucleotide. We found no evidence of contamination in mtDNA with *contamMix* (Fu *et al.* 2013b), and no evidence of X chromosome contamination with ANGSD (Korneliussen *et al.* 2014).

Table S.2. Contamination statistics for Burial 23-8

| **ANGSD SNPs** | **ANGSD MOM point estimate** | **ANGSD MOM Z-score** | **ANGSD MOM 95% CI truncated at 0** | **mt coverage** | **mt damage last base** | **mt consensus match** | **mt consensus match 95CI** |
| --- | --- | --- | --- | --- | --- | --- | --- |
| 480 | 0.007 | 1.376 | 0.000, 0.016 | 149.7 | 0.068 | 0.999266 | 0.994, 1.000 |

*Uniparental markers:* For mtDNA, consensus sequences were created with samtools and bcftools version 1.31 using majority rule and then using HaploGrep2 with Phylotree version 17; the consensus match was 0.994. The Y chromosome haplogroup was determined using the individual sequence data (BAM-file). We filtered out reads with mapping quality < 30 and bases with base quality < 30 and trimmed the first and last 2bp of each sequence to remove potential errors due to characteristic ancient DNA damage. We made a haplogroup determination based on the most derived mutation using the nomenclature of the International Society of Genetic Genealogy (ISOGG) (http://www.isogg.org) version ISOGG v15.73 (2020) notation using Yfitter (Jostins *et al.* 2014).

*Genetic Sex:* Genetic sex was determined by computing Y = the number of sequences overlapping SNPs on the Y chromosome (Y) and X = the number of sequences overlapping SNPs on the X chromosomes (X). We then computed the ratio R=Y/(X+Y). Using this calculation, genetic males have vales of R>0.35; Burial 23-8’s R=0.411.

**Demographic Modeling**

*Principal Component Analysis*: Principal component analysis was performed using smartPCA in EIGENSOFT (Patterson et al. 2012). We used the default parameters except for “lsqproject: YES” and “shrinkmode: YES” and performed PCA on modern Indigenous populations from the Americas including: Chane, Guarani, Huichol, Mixtec, Piapoco, Pima, Quechua, and Zapotec (Reich *et al.* 2012; Lazaridis *et al.* 2014; Skoglund *et al.* 2015; Barbieri *et al.* 2019). We then projected published ancient individuals (Raghavan *et al.* 2014, 2015; Malaspinas *et al.* 2014; Rasmussen *et al.* 2015; Kennett *et al.* 2017, 2022; Lindo *et al.* 2017; Moreno-Mayar *et al.* 2018a; b; Schroeder *et al.* 2018; Fuente *et al.* 2018; Scheib *et al.* 2018; Posth *et al.* 2018; Flegontov *et al.* 2019; Nieves-Colón *et al.* 2020; Nakatsuka *et al.* 2020a; b; Nägele *et al.* 2020; Bongers *et al.* 2020; Fernandes *et al.* 2021; Capodiferro *et al.* 2021; Popović *et al.* 2021; Tiesler *et al.* 2022) and Burial 23-8 on the principal components determined using present-day individuals.

*ADMIXTURE*: We used the ancient and modern populations above, along with European (America; Auton *et al.* 2015), Africa (Mbuti; Bergström *et al.* 2020) and South American (Surui, Karitiana; Bergström *et al.* 2020) populations to examine admixture. We first used PLINK2 (Chang et al. 2015) to prune the dataset using the –geno 0.7 option to remove all sites that had less than 70% of the samples with a called genotype. We then ran unsupervised ADMIXTURE (Alexander *et al.* 2009) with K=2 to 10 with 10 replicates for each K. We selected K=9 for figure 6 because of its usefulness for visual discrimination of ancestry components and because validation errors for K2-10 were not significantly different.

*f-statistics*: We used the *qp3pop* package in ADMIXTOOLS version 6.0 to compute *f_3_*-statistics. We computed outgroup *f_3_*-statistics of the form *f_3_(Pop1, Pop2; Mbuti)* to measure the shared genetic drift between Burial 23-8 (population 1) and Americas populations (population 2).

**Runs of Homozygosity Analyses**

We used *hapROH* (version 0.1a8; https://pypi.org/project/hapROH/) to identify runs of homozygosity (ROH) (Ringbauer *et al.* 2021).We used the 1000 Genomes Project haplotype panel as the reference panel with 5,008 global haplotypes. We then analyzed the ancient and present-day data of individuals with at least 300,000 SNPs covered to identify ROH longer than 4 cM. We used the default settings of hapROH for all analyses. All published individuals from the Americas that had high enough coverage to calculate RoH are provided in Supplementary Data 3 (Rasmussen *et al.* 2014, 2015; Scheib *et al.* 2018; Posth *et al.* 2018; Flegontov *et al.* 2019; Nakatsuka *et al.* 2020a; b; Nägele *et al.* 2020; Bongers *et al.* 2020; Fernandes *et al.* 2021; Tiesler *et al.* 2022; Kennett *et al.* 2022).

**Radiocarbon Dating**

As noted in the main manuscript, Burial 23-8 was buried beneath a post that had been dated through dendrochronology. Despite this, we felt it necessary to directly date Burial 23-8, especially considering the issues that have been reported with the dendrochronology of Paquimé (Dean & Ravesloot 1993; Lekson 2015). To obtain a date, we removed a small piece of bone from the same petrous (left) that was sampled for aDNA and sent it to the Pennsylvania State University AMS laboratory. Our first sampling attempt failed, but a second attempt from the left petrous was successful. The C and N isotope values are provided in Table S.3.

Table S.3. Radiocarbon data for Burial 23-8. PSU lab # PSUAMS-10865

| **^14^C age (BP)** | **±** | **δ^13^C (‰)** | **δ^15^N (‰)** | **%C** | **%N** | **C:N** |
| --- | --- | --- | --- | --- | --- | --- |
| 620 | 15 | -11.26727104 | 10.81560855 | 14.7303342 | 5.350976343 | 3.211636307 |

**Strontium Analysis**

Strontium analysis was performed at the Johnson Mass Spectrometry Laboratory at New Mexico State University, which has performed strontium ratio analysis for archaeological fauna remains from the same region (e.g., Semanko & Ramos 2022).

To minimize impact of destructive sampling, we used the remaining powder from the cochlea sampled from Burial 23-8 for Sr analysis. While there has been limited research on the lack of turnover of Sr in cochlea bone, what has been done (Harvig *et al.* 2014; Cavazzuti *et al.* 2019; Kootker & Laffoon 2022) showed that it does represent childhood strontium ratios, presumably for the same reason that cochlea bone is so good at preserving ancient DNA (Pinhasi *et al.* 2015). In any case, the young age of the individual means that there would have been insufficient time for the Sr ratio to have been changed from a birth location other than Paquimé to the value for Paquimé, especially since the strontium ratio was at the center of the Paquimé range. Moreover, a large sample of fauna bone strontium ratios from the site from the same study as the human tooth ratios bracket the local human ratios (Offenbecker 2018). This makes it clear that bone ratios are not subject to meaningful diagenesis in this locality and time frame. Thus, while the child’s strontium ratio comes from bone and not tooth enamel, like the other human samples, they are clearly comparable, and the child was both born and lived his short life at Paquimé.

Once in the Johnson laboratory the powder was cleaned, 50 milligrams of pre-powdered, bone mass was dissolved using doubly distilled, 6N hydrochloric acid (HCl). The powder was placed in a 15 ml Teflon container with approximately 5ml of doubly distilled 6N HCl. The sample was placed on a hotplate at ~180°C overnight.

After digestion, the sample was dried, redissolved in 1.0 ml of 2.5N HCl, and centrifuged. Approximately 0.5ml was loaded onto 22ml pyrex columns containing approximately 2ml of 200-400 mesh cation exchange resin. Doubly distilled and calibrated 2.5N HCl was used to obtain pure Sr following the procedures of Ramos (1992). Purified Sr solutions were then dried in preparation for loading and analyses using thermal ionization mass spectrometry (TIMS).

Sr was loaded onto pre-outgassed and clean rhenium filaments with phosphoric acid and tantalum oxide. Sr isotopes were analyzed using a VG Sector 54 thermal ionization mass spectrometer and five Faraday collectors in dynamic mode with ^88^Sr=3.0V. Sr isotopes were normalized to ^86^Sr/^88^Sr=01194 and corrected for any Rb present during the analysis. NBS987 Sr carbonate was 0.710275 ± 0.000010.

**Supplementary Table S.1—Analytical Processes and Results**

| **Information** | **Value** |
| --- | --- |
| Skeletal Code | 23-8 |
| Skeletal element | cochlea |
| 14C: Material used for radiocarbon measurement | XAD amino acids |
| 14C: Fraction Modern | 0.9255 ± 0.0017 |
| 14C: D^14^C (‰) | -74.5 ± 1.7 |
| 14C: Uncalibrated radiocarbon date (radiocarbon years BP) | 620 ± 15 |
| 14C: δ^13^C (‰) | -11.27 |
| 14C: δ^15^N (‰) | 10.82 |
| 14C: %C | 14.73 |
| 14C: %N | 5.35 |
| 14C: C:N ratio | 3.21 |
| 14C: Lab Code | PSUAMS-10865 |
| 14C: OxCal mu (years) | 604 |
| 14C: OxCal sigma (years) | 30 |
| 14C: 94.5% confidence interval for calibrated date | 1301-1397 calCE |
| 14C: Calibration Program | OxCal 4.4.2 and IntCal20 (Reimer *et al.* 2020; Bronk Ramsey 2021) |
| Genetic (methodology): Library ID | S22220.Y1.E2.L1 |
| Genetic (methodology): Individual ID | I22220 |
| Genetic (methodology): amount of powder used in DNA extraction (mg) | 39 |
| Genetic (methodology): Extraction Method | (Rohland *et al.* 2018); Dabney Buffer, silica beads, robotic |
| Genetic (methodology): Library preparation | double-stranded, partially UDG-treated |
| Genetic (methodology): amount of powder in library (mg) | 7.8 |
| Genetic (methodology): Library Preparation Method | (Rohland *et al.* 2015); silica coated magnetic beads and 7x volume PB (Qiagen) for cleanups, Bst2.0 in smaller volume, 100ul total PCR volume, higher primer concentration, SPRI cleanup of PCR |
| Genetic (methodology): Target enrichment performed | simultaneous enrichment of ~1.2M SNPs and mtDNA (Fu *et al.* 2013a, 2015) |
| Genetic (methodology): Sequencing platform for 1240k capture data | HiSeq X10: 2x101 reads, 2x7 bp to read out the indices |
| Genetic (shotgun sequencing of unenriched library): percentage mapping to the human reference genome hg19 | 0.038 |
| Genetic (shotgun sequencing of unenriched library): mean length of sequences | 53.9 |
| Genetic (enriched sequences mapping to the reference mtDNA genome sequences rsrs (Behar *et al.* 2012): average coverage | 149.7 |
| Genetic (enriched sequences mapping to the reference mtDNA genome sequences rsrs): mean sequence length | 58.4 |
| Genetic (enriched sequences mapping to the reference mtDNA genome sequences rsrs): fraction of C-to-T damage in last base | 0.068 |
| Genetic (enriched sequences mapping to the reference mtDNA genome sequences rsrs): match to consensus sequence 95% CI using contamMix version 1.0-12 (Fu *et al.* 2013a) | [0.994, 1.000] |
| Genetic (enriched sequences mapping to the reference mtDNA genome sequences rsrs): mtDNA haplogroup based on haplogrep 2 (Weissensteiner *et al.* 2016) | C1b |
| Genetic (enriched sequences mapping to targeted nuclear genomic positions): average coverage at 1.15M autosomal targeted SNPs | 0.454593 |
| Genetic (enriched sequences mapping to targeted nuclear genomic positions): targeted autosomal SNPs covered at least once | 395547 |
| Genetic (enriched sequences mapping to targeted nuclear genomic positions): mean sequence length | 63.1 |
| Genetic (enriched sequences mapping to targeted nuclear genomic positions): fraction of C-to-T damage in last base | 0.07 |
| Genetic (enriched sequences mapping to targeted nuclear genomic positions): targeted X chromosome SNPs covered at least once | 9273 |
| Genetic (enriched sequences mapping to targeted nuclear genomic positions): targeted Y chromosome SNPs covered at least once | 6461 |
| Genetic (enriched sequences mapping to targeted nuclear genomic positions): genetically determined sex | M |

**Works Cited**

Alexander, D.H., J. Novembre & K. Lange. 2009. Fast model-based estimation of ancestry in unrelated individuals. *Genome Research* 19: 1655–64. https://doi.org/10.1101/gr.094052.109.

Auton, A. et al. 2015. A global reference for human genetic variation. *Nature* 526: 68–74. https://doi.org/10.1038/nature15393.

Barbieri, C. et al. 2019. The Current Genomic Landscape of Western South America: Andes, Amazonia, and Pacific Coast. *Molecular Biology and Evolution* 36: 2698–2713. https://doi.org/10.1093/molbev/msz174.

Behar, D.M., M. van Oven, S. Rosset, M. Metspalu, E.-L. Loogväli, N.M. Silva, T. Kivisild, A. Torroni & R. Villems. 2012. A “Copernican” Reassessment of the Human Mitochondrial DNA Tree from its Root. *The American Journal of Human Genetics* 90: 675–84. https://doi.org/10.1016/j.ajhg.2012.03.002.

Bergström, A. et al. 2020. Insights into human genetic variation and population history from 929 diverse genomes. *Science* 367. American Association for the Advancement of Science: eaay5012. https://doi.org/10.1126/science.aay5012.

Bongers, J.L., N. Nakatsuka, C. O’Shea, T.K. Harper, H. Tantaleán, C. Stanish & L. Fehren-Schmitz. 2020. Integration of ancient DNA with transdisciplinary dataset finds strong support for Inca resettlement in the south Peruvian coast. *Proceedings of the National Academy of Sciences* 117. National Academy of Sciences: 18359–68. https://doi.org/10.1073/pnas.2005965117.

Bronk Ramsey, C. 2021. OxCal 4.4.4. https://c14.arch.ox.ac.uk/oxcalhelp/hlp_contents.html.

Capodiferro, M.R. et al. 2021. Archaeogenomic distinctiveness of the Isthmo-Colombian area. *Cell* 184: 1706-1723.e24. https://doi.org/10.1016/j.cell.2021.02.040.

Cavazzuti, C., R. Skeates, A.R. Millard, G. Nowell, J. Peterkin, M.B. Brea, A. Cardarelli & L. Salzani. 2019. Flows of people in villages and large centres in Bronze Age Italy through strontium and oxygen isotopes. *PLOS ONE* 14. Public Library of Science: e0209693. https://doi.org/10.1371/journal.pone.0209693.

Dabney, J. et al. 2013. Complete mitochondrial genome sequence of a Middle Pleistocene cave bear reconstructed from ultrashort DNA fragments. *Proceedings of the National Academy of Sciences* 110: 15758–63. https://doi.org/10.1073/pnas.1314445110.

Dean, J.S. & J.C. Ravesloot. 1993. The Chronology of Cultural Interaction in the Gran Chichimeca, in A.I. Woosley & J.C. Ravesloot (ed.) *Culture and Contact: Charles C.D. Di Peso’s Gran Chichimeca*: 83–103 (Amerind Foundation New World Studies Series 2). Albuquerque, NM: University of New Mexico Press.

Fernandes, D.M. et al. 2021. A genetic history of the pre-contact Caribbean. *Nature* 590. Nature Publishing Group: 103–10. https://doi.org/10.1038/s41586-020-03053-2.

Flegontov, P. et al. 2019. Palaeo-Eskimo genetic ancestry and the peopling of Chukotka and North America. *Nature* 570: 236–40. https://doi.org/10.1038/s41586-019-1251-y.

Fu, Q., M. Meyer, X. Gao, U. Stenzel, H.A. Burbano, J. Kelso & S. Paabo. 2013a. DNA analysis of an early modern human from Tianyuan Cave, China. *Proceedings of the National Academy of Sciences* 110: 2223–27. https://doi.org/10.1073/pnas.1221359110.

Fu, Q. et al. 2013b. A Revised Timescale for Human Evolution Based on Ancient Mitochondrial Genomes. *Current Biology* 23: 553–59. https://doi.org/10.1016/j.cub.2013.02.044.

—. 2015. An early modern human from Romania with a recent Neanderthal ancestor. *Nature* 524: 216–19. https://doi.org/10.1038/nature14558.

Fuente, C. de la et al. 2018. Genomic insights into the origin and diversification of late maritime hunter-gatherers from the Chilean Patagonia. *Proceedings of the National Academy of Sciences* 115: E4006–12. https://doi.org/10.1073/pnas.1715688115.

Harvig, L., K.M. Frei, T.D. Price & N. Lynnerup. 2014. Strontium Isotope Signals in Cremated Petrous Portions as Indicator for Childhood Origin. *PLOS ONE* 9. Public Library of Science: e101603. https://doi.org/10.1371/journal.pone.0101603.

Jostins, L., Y. Xu, S. McCarthy, Q. Ayub, R. Durbin, J. Barrett & C. Tyler-Smith. 2014. YFitter: Maximum likelihood assignment of Y chromosome haplogroups from low-coverage sequence data. *arXiv:1407.7988 [q-bio]*. http://arxiv.org/abs/1407.7988.

Kennett, D.J. et al. 2017. Archaeogenomic evidence reveals prehistoric matrilineal dynasty. *Nature Communications* 8: 14115. https://doi.org/10.1038/ncomms14115.

—. 2022. South-to-north migration preceded the advent of intensive farming in the Maya region. *Nature Communications* 13: 1530. https://doi.org/10.1038/s41467-022-29158-y.

Kootker, L.M. & J.E. Laffoon. 2022. Assessing the preservation of biogenic strontium isotope ratios (87Sr/86Sr) in the pars petrosa ossis temporalis of unburnt human skeletal remains: A case study from Saba. *Rapid Communications in Mass Spectrometry* 36: e9277. https://doi.org/10.1002/rcm.9277.

Korneliussen, T.S., A. Albrechtsen & R. Nielsen. 2014. ANGSD: Analysis of Next Generation Sequencing Data. *BMC Bioinformatics* 15: 356. https://doi.org/10.1186/s12859-014-0356-4.

Lazaridis, I. et al. 2014. Ancient human genomes suggest three ancestral populations for present-day Europeans. *Nature* 513: 409–13. https://doi.org/10.1038/nature13673.

Lekson, S.H. 2015. *The Chaco Meridian: One Thousand Years of Political and Religious Power in the Ancient Southwest*. Second edition. Lanham ; Boulder ; New York ; London: Rowman & Littlefield Publishers.

Lindo, J. et al. 2017. Ancient individuals from the North American Northwest Coast reveal 10,000 years of regional genetic continuity. *Proceedings of the National Academy of Sciences* 114: 4093–98. https://doi.org/10.1073/pnas.1620410114.

Malaspinas, A.-S. et al. 2014. Two ancient human genomes reveal Polynesian ancestry among the indigenous Botocudos of Brazil. *Current Biology* 24: R1035–37. https://doi.org/10.1016/j.cub.2014.09.078.

Mathieson, I. et al. 2015. Genome-wide patterns of selection in 230 ancient Eurasians. *Nature* 528: 499–503. https://doi.org/10.1038/nature16152.

Moreno-Mayar, J.V. et al. 2018a. Terminal Pleistocene Alaskan genome reveals first founding population of Native Americans. *Nature* 553: 203–7. https://doi.org/10.1038/nature25173.

—. 2018b. Early human dispersals within the Americas. *Science* 362: eaav2621. https://doi.org/10.1126/science.aav2621.

Nägele, K. et al. 2020. Genomic insights into the early peopling of the Caribbean. *Science* 369. American Association for the Advancement of Science: 456–60. https://doi.org/10.1126/science.aba8697.

Nakatsuka, N. et al. 2020a. A Paleogenomic Reconstruction of the Deep Population History of the Andes. *Cell* 181: 1131-1145.e21. https://doi.org/10.1016/j.cell.2020.04.015.

—. 2020b. Ancient genomes in South Patagonia reveal population movements associated with technological shifts and geography. *Nature Communications* 11: 3868. https://doi.org/10.1038/s41467-020-17656-w.

Nieves-Colón, M.A. et al. 2020. Ancient DNA Reconstructs the Genetic Legacies of Precontact Puerto Rico Communities. (ed.)C. Mulligan *Molecular Biology and Evolution* 37: 611–26. https://doi.org/10.1093/molbev/msz267.

Pinhasi, R. et al. 2015. Optimal Ancient DNA Yields from the Inner Ear Part of the Human Petrous Bone. (ed.)M.D. Petraglia *PLOS ONE* 10: e0129102. https://doi.org/10.1371/journal.pone.0129102.

Popović, D. et al. 2021. Ancient genomes reveal long-range influence of the pre-Columbian culture and site of Tiwanaku. *Science Advances* 7. American Association for the Advancement of Science: eabg7261. https://doi.org/10.1126/sciadv.abg7261.

Posth, C. et al. 2018. Reconstructing the Deep Population History of Central and South America. *Cell* 175: 1185-1197.e22. https://doi.org/10.1016/j.cell.2018.10.027.

Raghavan, M. et al. 2014. The genetic prehistory of the New World Arctic. *Science* 345: 1255832–1255832. https://doi.org/10.1126/science.1255832.

—. 2015. Genomic evidence for the Pleistocene and recent population history of Native Americans. *Science* 349: aab3884–aab3884. https://doi.org/10.1126/science.aab3884.

Ramos, F.C. 1992. Isotope Geology of the Metamorphic Core of the Central Grouse Creek Mountains, Box Elder County, Utah. MA Thesis, University of California, Los Angeles.

Rasmussen, M. et al. 2014. The genome of a Late Pleistocene human from a Clovis burial site in western Montana. *Nature* 506: 225–29. https://doi.org/10.1038/nature13025.

—. 2015. The ancestry and affiliations of Kennewick Man. *Nature*. http://www.nature.com/doifinder/10.1038/nature14625. https://doi.org/10.1038/nature14625.

Reich, D. et al. 2012. Reconstructing Native American population history. *Nature* 488. Nature Publishing Group: 370–74. https://doi.org/10.1038/nature11258.

Reimer, P.J. et al. 2020. The IntCal20 Northern Hemisphere Radiocarbon Age Calibration Curve (0–55 cal kBP). *Radiocarbon* 62. Cambridge University Press: 725–57. https://doi.org/10.1017/RDC.2020.41.

Ringbauer, H., J. Novembre & M. Steinrücken. 2021. Parental relatedness through time revealed by runs of homozygosity in ancient DNA. *Nature Communications* 12: 5425. https://doi.org/10.1038/s41467-021-25289-w.

Rohland, N., E. Harney, S. Mallick, S. Nordenfelt & D. Reich. 2015. Partial uracil-DNA-glycosylase treatment for screening of ancient DNA. *Philosophical Transactions of the Royal Society of London. Series B, Biological Sciences* 370: 20130624. https://doi.org/10.1098/rstb.2013.0624.

Rohland, N., I. Glocke, A. Aximu-Petri & M. Meyer. 2018. Extraction of highly degraded DNA from ancient bones, teeth and sediments for high-throughput sequencing. *Nature Protocols* 13: 2447–61. https://doi.org/10.1038/s41596-018-0050-5.

Scheib, C.L. et al. 2018. Ancient human parallel lineages within North America contributed to a coastal expansion. *Science* 360: 1024–27. https://doi.org/10.1126/science.aar6851.

Schroeder, H. et al. 2018. Origins and genetic legacies of the Caribbean Taino. *Proceedings of the National Academy of Sciences* 115: 2341–46. https://doi.org/10.1073/pnas.1716839115.

Semanko, A. & F.C. Ramos. 2022. Teaching an Old Dog New Tricks: Implications for Isotopic Studies of Southwest Dogs. *KIVA* 88: 327–46. https://doi.org/10.1080/00231940.2022.2036911.

Skoglund, P., S. Mallick, M.C. Bortolini, N. Chennagiri, T. Hünemeier, M.L. Petzl-Erler, F.M. Salzano, N. Patterson & D. Reich. 2015. Genetic evidence for two founding populations of the Americas. *Nature*. http://www.nature.com/doifinder/10.1038/nature14895. https://doi.org/10.1038/nature14895.

Tiesler, V. et al. 2022. Life and death in early colonial Campeche: new insights from ancient DNA. *Antiquity* 96. Cambridge University Press: 937–54. https://doi.org/10.15184/aqy.2022.79.

Weissensteiner, H., D. Pacher, A. Kloss-Brandstätter, L. Forer, G. Specht, H.-J. Bandelt, F. Kronenberg, A. Salas & S. Schönherr. 2016. HaploGrep 2: mitochondrial haplogroup classification in the era of high-throughput sequencing. *Nucleic Acids Research* 44: W58-63. https://doi.org/10.1093/nar/gkw233.
